# Mono-valent salt corrections for RNA secondary structures in the ViennaRNA package

**DOI:** 10.1186/s13015-023-00236-0

**Published:** 2023-07-29

**Authors:** Hua-Ting Yao, Ronny Lorenz, Ivo L. Hofacker, Peter F. Stadler

**Affiliations:** 1 Department of Theoretical Chemistry, University of Vienna, Währinger Straße 17, 1090 Vienna, Austria; 2 Research Group Bioinformatics and Computational Biology, Faculty of Computer Science, University of Vienna, Währingerstraße 29, 1090 Vienna, Austria; 3 Bioinformatics Group, Department of Computer Science, and Interdisciplinary Center for Bioinformatics, Universität Leipzig, Härtelstraße 16–18, 04107 Leipzig, Germany; 4 Competence Center for Scalable Data Services and Solutions Dresden/Leipzig, Interdisciplinary Center for Bioinformatics, German Centre for Integrative Biodiversity Research (iDiv), and Leipzig Research Center for Civilization Diseases, Universität Leipzig, Augustusplatz 12, 04107 Leipzig, Germany; 5 Max Planck Institute for Mathematics in the Sciences, Inselstraße 22, 04109 Leipzig, Germany; 6 Facultad de Ciencias, Universidad National de Colombia, Sede Bogotá, Ciudad Universitaria, 111321 Bogotá, D.C., Colombia; 7 Santa Fe Institute, 1399 Hyde Park Rd., NM87501 Santa Fe, USA

**Keywords:** RNA secondary structure, Salt concentration, Debye-Hückel potential

## Abstract

**Background:**

RNA features a highly negatively charged phosphate backbone that attracts a cloud of counter-ions that reduce the electrostatic repulsion in a concentration dependent manner. Ion concentrations thus have a large influence on folding and stability of RNA structures. Despite their well-documented effects, salt effects are not handled consistently by currently available secondary structure prediction algorithms. Combining Debye-Hückel potentials for line charges and Manning’s counter-ion condensation theory, Einert et al. (Biophys J 100: 2745-2753, 2011) modeled the energetic contributions of monovalent cations on loops and helices.

**Results:**

The model of Einert et al. is adapted to match the structure of the dynamic programming recursion of RNA secondary structure prediction algorithms. An empirical term describing the salt dependence of the duplex initiation energy is added to improve co-folding predictions for two or more RNA strands. The slightly modified model is implemented in the ViennaRNA package in such way that only the energy parameters but not the algorithmic structure is affected. A comparison with data from the literature show that predicted free energies and melting temperatures are in reasonable agreement with experiments.

**Conclusion:**

The new feature in the ViennaRNA package makes it possible to study effects of salt concentrations on RNA folding in a systematic manner. Strictly speaking, the model pertains only to mono-valent cations, and thus covers the most important parameter, i.e., the NaCl concentration. It remains a question for future research to what extent unspecific effects of bi- and tri-valent cations can be approximated in a similar manner.

**Availability:**

Corrections for the concentration of monovalent cations are available in the ViennaRNA package starting from version 2.6.0.

**Supplementary Information:**

The online version contains supplementary material available at 10.1186/s13015-023-00236-0.

## Introduction

Nucleic acids are highly negatively charged molecules since their phosphate backbone carries one negative charge per nucleotide. Structure formation brings these charges into close proximity and thus incurs substantial electrostatic penalties on the secondary and tertiary structure level. Nucleic acid–ion interactions also provide large interaction energies and therefore contribute decisively to the folding of RNA and DNA and to their interactions with ligands and macromolecule partners [1]. Counter-ions reduce the electrostatic repulsion of the backbone. Cation concentrations determine the extent of this “charge screening” and thus strongly influence RNA folding. Indeed, many functional RNAs will not fold under low salt conditions [2], and experimental investigations of the thermodynamics of RNA folding are mostly confined to high salt conditions. Energy parameters for RNA secondary predictions likewise pertain to 1M sodium concentrations, more precisely 1.021 M while taking all $\hbox {Na}^{+}$ ions in the buffer into account [3]. Although the importance of counter-ions for the RNA folding is well known, ion concentration, in contrast to temperature, is not a tunable parameter in most of the currently available RNA secondary structure prediction tools. Although NUPACK [4] and UNAfold [5] offer a corresponding option, they do not handle salt effects in a consistent manner. The empirical salt corrections (derived from DNA for $\hbox {Na}^{+}$ [6] and $\hbox {Mg}^{+}$ [7]) offered by these tools pertain to stacking energies contributions only and neglect the loop energies due to insufficient empirical data. Loops, however, are subject to salt-dependent effects in a similar energy range as we shall see below. While temperature dependence is conceptually straightforward and can be easy modeled by splitting free energy contributions into enthalpic and entropic contributions [8], the energetics of ion–nucleic acid interactions are much more difficult to understand.

Cations affect RNA structure in two different ways. The electrostatic stabilization of the structure due to charge screening is at least conceptually independent of the chemical nature and charge of the individual cations. In addition, divalent cations and in particular $\hbox {Mg}^{+}$ can also strongly bind to specific, chelating sites [9, 10]. Quantitative salt effects on RNA folding have been studied extensively over the last decades, see [11] for a study that summarized much of the pertinent earlier literature. In the absence of a well-founded theoretical model, most authors resorted to describing the salt-dependence of RNA folding by means of simple heuristic function fitting the effects of changes in the sodium concentration on the free energy of folding or a melting temperature. Such empirical fits, however, are limited to handling salt effects close to standard conditions, and an approach that explains the functional form of salt effects is clearly preferable.

If three-dimensional structures are known, the nonlinear Poisson-Boltzmann equation can be solved to obtain electrostatic potentials of RNA molecules in solution [12–14]. This approach, however, appears to be too detailed to derive a practically manageable parametrization of salt effects at the level of secondary structure prediction algorithms. In order to handle counter-ions in RNA secondary structure prediction algorithms, the effects must be attributed to individual bases, base pairs, or loops (including the stacking of two consecutive base pairs). This is necessary because secondary structure prediction algorithms operate on these combinatorial substructures [15]. This, in particular, precludes models that explicitly require a detailed geometric description of three-dimensional structure of an RNA. Ensembles of 3D structure models can be used, however, to estimate cation effects on loops and helices empirically as a alternative to wet-lab experiments [16, 17]. A coarse grained model representing each nucleotide by two virtual bonds (C$_4$-P and P-C$_4$) [18] and tightly-bound ion theory [19] accounting for strongly correlated multi-valent ions was employed to sample loop conformations in the presence of both $\hbox {Na}^{+}$ and $\hbox {Mg}^{+}$. Empirical expressions for electrostatic helix [16] and loop [17] energy contributions were extracted from these simulations.

To derive a suitably simple model, Einert and Netz [20] proposed to represent the RNA backbone as a charged polymer that interacts by means of a Debye-Hückel potential [21] and treats single-stranded regions as freely jointed chains [22]. The non-linear screening effect of monovalent cations is incorporated using Manning’s approach to counter-ion condensation [23]. The formulation for loop contribution was originally developed to understand the salt-dependent modulation of nucleosomal structures [24]. The two strands of a helix are modeled as parallel rods that again interact via a Debye-Hückel potential governed by the screening length. Here, the theory yields a per-position contribution that is independent of sequence features and the position within the helix [20]. While the theory makes several approximations it has been shown by its authors to reproduce experimental data quite well. It also has the advantage that it has no free parameters other than well known generic geometric characteristics of nucleic acid 3D structures such as distances between nucleotides or the planes of stacked pairs. In contrast to empirical salt corrections derived from measurements or simulations, the theory has the advantage of behaving reasonably also outside the regime of available measurements. Of course, the secondary structure based approaches have significant limitations. In particular, they are by design not suitable to model the site-specific binding of chelated $\hbox {Mg}^{+}$, which in some cases is known to be crucial for tertiary structure formation and RNA function [25].

Here we implement in the ViennaRNA package [26] the Debye-Hückel/Manning model derived in [20], which captures the energetic effects of electrostatic interactions of mono-valent cations with structured RNA. In the following theory section we briefly review the features of the energy model and show that it can be brought into a form where only the energy parameters but not the dynamic programming recursions are modified. We then evaluate the model on a collection of empirical data from the literature to show that use of this type of salt correction has a significant beneficial effect.

## Theory

The model of [20] considers loops as freely jointed charged chains and helices as parallel rods interacting via Debye-Hückel potentials and uses Manning’s framework to model counter-ions condensation. This results in distinct types of correction terms from loops and helices, which we describe separately in the following. In either case we focus on how the salt correction terms are incorporated into the dynamic programming schemes for RNA secondary structure prediction. As we shall see, the salt corrections can be phrased in such a way that they exclusively effect the energy parameters. The folding algorithms therefore remain unchanged. Owing to the architecture of the ViennaRNA package, it is therefore possible to handle salt effects exclusively as a pre-processing energy parameter set. The presentation below focuses on RNA, since secondary structure predictions (beyond perfect duplexes) are of practical interest mostly for RNA. The theory, however, applies equally to DNA secondary structures.

### Salt corrections for loops

The electrostatic free energy contribution for a loop comprising *L* backbone bonds can be written, at the level of the Debye-Hückel approximation, in the form [24]:

1 $${\mathcal{G}}_{u} (L) = RT\frac{{\ell _{B} }}{\kappa }\tau _{{ss}}^{2} \Phi (y)$$

with $y=\kappa l_{ss}L$ where $l_{ss}$ is the length of a single stranded RNA backbone bond (phosphate-to-phosphate distance), $\ell _B$ is the Bjerrum length and $\kappa ^{-1}$ is the Debye screening length, which depends on the ionic strength, and $\tau _{ss}=\min (1/l_{ss},1/\ell _Bz_c)$ accounts for the nonlinear electrostatic effects. For monovalent ions, the ionic strength equals the salt concentration $\rho$ and thus we have $\kappa =\kappa (\rho )$. Following [24], $\Phi$ is given by

2 $$\begin{aligned} \begin{aligned} \Phi (y)&= y\ln y + y\left( \gamma -\ln (\pi /2)\right) - \frac{y^2}{2}\, {}_{1}F_{2}\left( \begin{matrix} 1/2 \\ 1,\, 3/2 \end{matrix}\,\bigg \vert \, \frac{y^2}{4\pi ^2}\right) \\&\frac{y^3}{2\pi ^2}\, {}_{2}F_{3}\left( \begin{matrix} 1,\, 1 \\ 3/2,\, 3/2,\, 2 \end{matrix} \,\bigg \vert \, \frac{y^2}{4\pi ^2}\right) + 1 -\exp (-y)+y\Gamma (0,y) \end{aligned} \end{aligned}$$

Here, ${}_{1}F_{2}$ and ${}_{2}F_{3}$ are generalized hypergeometric functions and $\Gamma (0,y)$ is the incomplete gamma function. Using ${}_{1}F_{2}(\dots ,0)={}_{2}F_{3}(\dots ,0)=1$, and $y\ln y\rightarrow 0$ for $y\rightarrow 0$, we observe that $\Phi (y)=0$. Eq. (A8) in [24] gives an expansion for small *y* of form $\Phi (y)/y= (1-\ln (\pi /2))+O(y^2)$, where the $\ln y$ term and the logarithmic divergence of $\Gamma (0,y)$ cancel. Thus, $\Phi (y)$ increases linearly with *y*. Since both *y* and $\kappa$ are proportional to $\sqrt{\rho }$, the $\rho$-dependence cancels and ${\mathcal {G}}_u(L)$ approaches *L* times a constant for $\rho \rightarrow 0$.

Since ${\mathcal {G}}_u(L)$ describes the salt-dependent electrostatic effects on loops, this term is already included in the empirical energy parameters of the RNA standard model for the standard conditions of $T=37^{\circ }C$ and 1 M sodium concentration. Writing $\widehat{{\mathcal {G}}}_u(L)$ for the values on Eq. (1) at standard conditions, allows us to write the salt correction of a given loop as

3 $$\begin{aligned} g_{\text {u}}(L,\rho ):= {\mathcal {G}}_u(L)-\widehat{{\mathcal {G}}}_u(L) \end{aligned}$$

The ViennaRNA package quantifies the length of a loop by the number *m* of unpaired nucleotides rather than the number of bonds. For hairpin loops we have $L=m+1$, while $L=m+2$ for interior loops. In multi-loops we have $L=m+q+1$, where *q* is the degree (number of branches) of the multi-loop. Setting $q=0$ for hairpin loops and $q=1$ for interior loops, the appropriate salt correction for a loop is therefore $g_{\text {u}}(m+q+1,\rho )$.

**Fig. 1:**
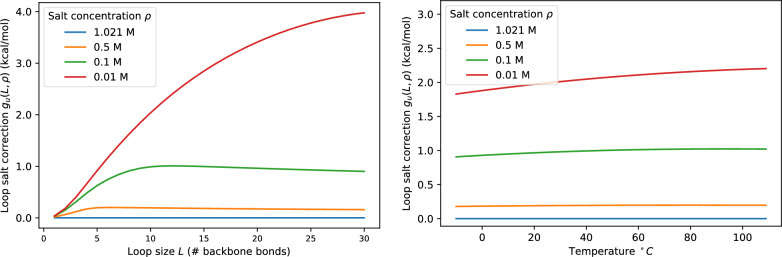
Loop salt correction $g_{\text {u}}(L,\rho )$ as a function of loop size $L=m+q+1$ (left) and as a function of temperature (right) for a fixed loop at size $L=10$ for different salt concentrations $\rho$

As seen in Fig. 1, the salt correction, as expected, increases while decreasing the salt concentration contributing to the destabilization of the structure in a low salt environment. In the usual temperature range, the plot shows that loop correction is close to a constance with a slight increase at low concentration.

### Linear approximation for multi-branch Loop

The free energy of a loop asymptotically depends on the logarithm of its length [27], see also [28] for a recent review. For multi-loops, however, this behavior is usually approximated by a linear function

4 $$\begin{aligned} E_{ml} = \alpha + \beta q + \gamma m \end{aligned}$$

for the sake of computational efficiency. The parameters $\alpha$, $\beta$, and $\gamma$ are the energy cost for having, respectively, the closing pair, branch, and unpaired base in a multi-loop. The reason for this linearisation is that recursions implementing a non-linear dependence of the multi-loop contribution on *m* or $m+q$ make it necessary to store terms pertaining to substructures delimited by the closing pair of multi-loop of size *m*. This leads to a cubic-memory and quartic-time algorithm, which is infeasible for larger RNA molecules [29]. The linear approximation is further motivated by the empirical observation that models with logarithmic dependence are outperformed by the linear approximation in terms of accuracy of structure prediction [29].

In order to handle multi-loops without abandoning the memory-efficient multi-loop decomposition of the standard RNA energy model [3], it is necessary to retain the linear form of the multi-loop terms also in the presence of salt corrections. This implies that the salt-correction itself must also be linear in both *q* and *m*. Using $L=m+q+1$ this implies that the salt correction must be of the form

5 $$\begin{aligned} g_{\text {u}}(m,q,\rho ) \approx a_0(\rho ) + a_1(\rho ) L \end{aligned}$$

where $a_0$ and $a_1$ are the parameters of the linear approximation of $g_{\text {u}}(L,\rho )$. The correction therefore amounts to adding $a_0+a_1$ to the closing pair term $\alpha$ of the multi-loop and $a_1$ to both, each unpaired base $\gamma$ and each component $\beta$ of the multi-loop. The salt-dependent multi-loop model thus reads:

6 $$\begin{aligned} E_{ml} = (\alpha + a_0(\rho ) + a_1(\rho )) + (\beta + a_1(\rho ))q + (\gamma + a_1(\rho ))m \end{aligned}$$

**Fig. 2:**
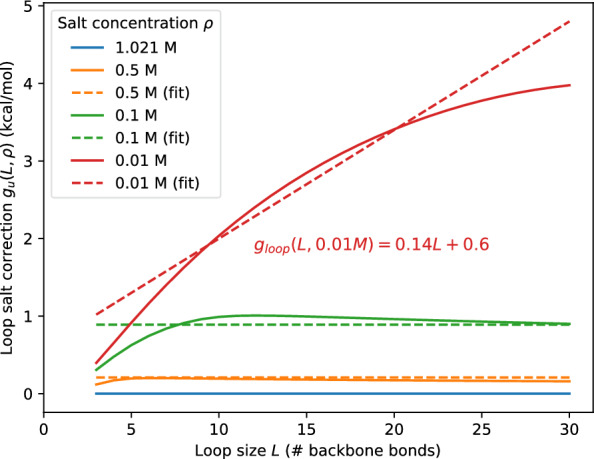
Loop salt correction (solid) and linear approximation (dashed) in the function of loop size *L* at different salt concentration

In order to fit the coefficients $a_0$ and $a_1$ appearing in the multi-loop parameters in practice, we first investigated their size distribution in samples of minimum free energy (MFE) structures of $5~000$ random sequences for different RNA sizes, as well as in the natural RNA structures recorded in the RNA STRAND database [30], see Additional file 1: Fig. S1. Although multi-loops with size $L=3$ exist, very short multi-loops are rare. We argue that inaccuracies in these rare cases are likely acceptable. Very short loops presumable are also subject to specifically constrained three-dimensional structures and thus follow the model only to a crude approximation in the first place. In the current implementation, we use the loop size range $L\in [6,24]$ to obtain linear fits for $a_0$ and $a_1$ from $g_{\text {u}}(L,\rho )$, see Fig. 2 for salt correction and their linear approximations. In general, the fit over-corrects for very small loops and, at low salt concentrations, also for very large loops. The maximal fitting errors are on the order of $1 \text { kcal/mol}$, which is still within the ball-park of the rather large inaccuracies expected for multi-loop energies.

### Salt corrections for stacked base pairs

Describing the backbones of stacked pairs as rods with distance of $d=20$ Å interacting via a Debye-Hückel potential with screening length $1/\kappa$ yields the following electrostatic energy for the interaction of a nucleotide with the other strand [20]:

7 $$\begin{aligned} {\mathcal {G}}_p = 2RT \tau ^2_{ds}l_{ds}\ell _B K_0(\kappa d) \end{aligned}$$

Here the charge density $\tau _{ds}=\min (1/l_{ds},1/(\ell _Bz_c))$ is again estimated according to Manning’s counterion condensation theory [23]. The length parameter $l_{ds}$ is the helical rise per base pair and $z_c=1$ is the charge of the cation. $K_0$ denotes the zeroth-order modified Bessel function of the 2nd kind, see e.g. [31] and Additional file 1: Fig. S2. Since $K_0(z)$ diverges like $-\ln z$ for $z\rightarrow 0$, and $\kappa \sim \sqrt{\rho }$, ${\mathcal {G}}_p$ diverges logarithmically for vanishing salt concentrations.

As in the case of loops, these electrostatic effects are already included in the empirical energy parameters for standard conditions. The relevant salt correction thus is given by the difference between the values ${\mathcal {G}}_p$ at the current conditions and standard conditions.

8 $$\begin{aligned} g_p:= {\mathcal {G}}_p - \widehat{{\mathcal {G}}}_p \end{aligned}$$

**Fig. 3:**
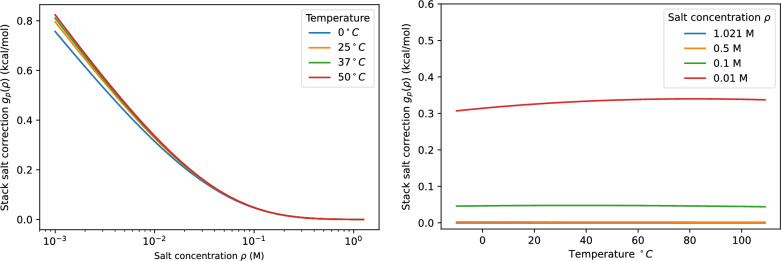
Salt correction for a stacked pair as a function of salt concentration (left) and temperature (right)

Figure 3 shows the dependence of the position-wise salt correction for stacking energies as function of salt concentration and temperature. Similar to the loop correction, the stack correction is close to a constant in the usual temperature range for a given salt concentration.

### Salt corrections for duplex initialization

The formation of a double strand from two RNA molecules in solution is associated with an additional initialization energy $E_{\text {init}}$ in the Turner energy model. One expects that duplex formation becomes more difficult due to electrostatic repulsion at low salt concentrations. The initialization energy thus should also depend on $\rho$. In addition, the distance between two single strands changes during formation, which was neglected in the theory as discussed in [20]. Indeed, as we will see later in the experimental data comparison section, the duplex free energies are systematically overestimated compared to the experimental data.

**Fig. 4:**
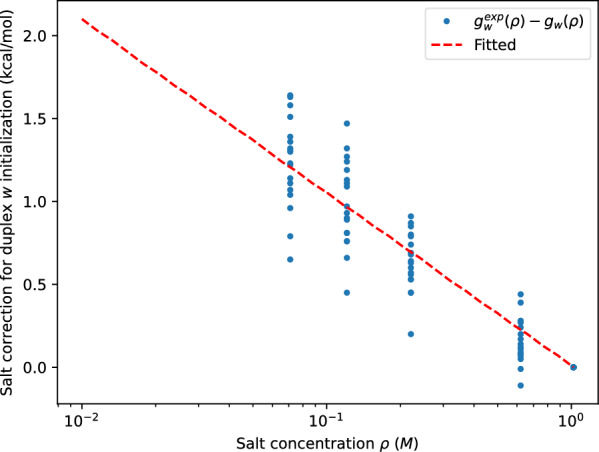
Salt correction for duplex initialization fitted (red) from the difference between experimental and predicted duplex salt correction (blue)

Due to the lack of theoretical support, we propose a salt correction for duplex initialization $g_{\text {init}}(\rho )$ derived from the prediction and the experimental data taken from [11]. Let $g_w(\rho )$ and $g_w^{\text {exp}}(\rho )$ be the predicted and experimental salt correction at concentration $\rho$ from the standard condition for a give duplex $w$. Fitting the difference $g_w^{\text {exp}}(\rho )-g_w(\rho )$ of $18$ duplexes at four non-standard sodium concentrations yields, as plotted in Fig. 4, the salt corrections for duplex initialization

9 $$\begin{aligned} g_{\text {init}}(\rho ) = a \ln (\frac{\rho }{\rho _0}) \end{aligned}$$

with $a= -0.45324$ kcal/mol for RNA and $a=-0.58389$ kcal/mol for DNA.

The nature of the initialization term *a* is not quite clear from the literature. On the one hand, it is argued as an entropic contribution in [32]. On the other hand, it is included with a large enthalpic contribution in recent versions of the standard energy model [3]. Given that the temperature dependence of the salt corrections for both stacking and loop is small, we feel justified in assuming *a* to be temperature-independent. At present, there are no data to test this assumption.

## Implementation in ViennaRNA

The extension of ViennaRNA provides access to four user-defined parameters: the concentration of the monovalent cation $\rho$, the bounds $L_1$ and $L_2$ delimiting the interval of loop length that is used to fit the two multiloop parameters $a_0$ and $a_1$ for given salt concentration, and the user-provided salt correction *g* for duplex initialization. Default values are $L_1=6$, $L_2=24$, salt concentration $\rho =1.021$ M, and $g=99999$, indicating no value is provided.

On the command line, a new option –salt provides access to the salt corrections in all interactive programs of the ViennaRNA package. Internally, the relevant parameters are appended in the model object vrna_md_s as salt for $\rho$, saltMLLower and saltMLUpper for $L_1$ and $L_2$, and saltDPXInit for $g$. For the salt concentration, ViennaRNA assumes the standard conditions of the Turner energy model, i.e., $\rho =1.021$ M. Thus no salt corrections apply by default. A detailed description of the API in the ViennaRNA library is available at https://www.tbi.univie.ac.at/RNA/ViennaRNA/refman/index.html.

If a different concentration $\rho$ is requested, first the value of $g_{\text {p}}(\rho )$ for the stack and the array of values $g_{\text {u}}(L,\rho )$ for loop of different size $L\in [1,31]$ is computed. Note that these energy contributions depend on the temperature *T* and thus are recomputed if the user sets a different temperature. In addition, the use of duplex initialization salt correction $g_{\text {init}}(\rho )$ for duplex is turned off if users set saltDPXInit to zero.

The array $g_u$ is then used to determine $a_0(\rho )$ and $a_1(\rho )$ by linear regression. Subsequently, the free energy parameters are set as sums of the default values *E*(0) for the given temperature and the salt corrections:

10 $$\begin{aligned} \begin{aligned} E^{bp}&= E^{bp}(0) + g_{\text {p}}(\rho ) \\ E^{HL}_{m}&= E^{HL}_m(0) + g_{\text {u}}(m+1,\rho ) \\ E^{IL}_{m}&= E^{IL}_m(0) + g_{\text {u}}(m+2,\rho ) \\ \alpha&= \alpha (0) + a_0(\rho ) + a_1(\rho ) \\ \beta&= \beta (0) + a_1(\rho ) \\ \gamma&= \gamma (0) + a_1(\rho ) \\ E_{\text {init}}&= E_{\text {init}} + g_{\text {init}}(\rho ) \\ \end{aligned} \end{aligned}$$

Here $E^{bp}$, $E^{HL}_{m}$, $E^{HL}_{m}$, and $E_{\text {init}}$ refer to *all* parameters for stacked pairs, hairpin loops of length *m*, interior loops of length *m*, and duplex initialization, respectively. Dangling end and coaxial stacking contributions, on the other hand, remain unchanged.

The Bessel functions $K_0$ is computed as in scipy, which in turn used the cephes mathematical function library described in [33]. The function $\Phi$ in loop salt correction can be approximated as given in [20]:

11 $$\begin{aligned} \begin{aligned} \Phi (y)&= y\ln y + y(\gamma - \ln (\pi /2)) + y\frac{(2\pi )^6}{y^6+(2\pi )^6} \left( \frac{y^4}{36\pi ^4} - \frac{y^3}{24\pi ^2} + \frac{y^2}{2\pi ^2} - \frac{y}{2} \right) \\ \quad+ \left( 1-\frac{(2\pi )^6}{y^6+(2\pi )^6}\right) \left( y(\log (2\pi ) -1.96351) - y\log y \right) + \left( 1-e^{-y}\right) + y\Gamma (0,y) \end{aligned} \end{aligned}$$

### Parameters

The key physical parameter containing the salt correction terms are the Debye screening length $\kappa ^{-1}$. It is convenient to express $\kappa ^{-1}$ in terms of Bjerrum length $\ell _B$ and the ionic strength *I*:

12 $$\begin{aligned} & \ell _B= \dfrac{e^2}{4\pi k_BT\epsilon _0\epsilon _r(T)} \approx \left( 167092.53 \text{\AA K} \right) \frac{1}{T\epsilon _r(T)} \\& \kappa ^{-1}= \sqrt{\dfrac{k_BT\epsilon _0\epsilon _r(T)}{2N_Ae^2I}} = \dfrac{1}{\sqrt{8\pi N_A}}\dfrac{1}{\sqrt{\ell _B I}} \approx \left( 8.1285 \text{\AA }^{\frac{3}{2}}\text {mol}^{\frac{1}{2}}\text {L}^{-\frac{1}{2}}\right) \frac{1}{\sqrt{\ell _B I}} \end{aligned}$$

The values of physical constants appearing in these expressions are taken from CODATA [34] and listed in Additional file 1: Table S1. The salt concentration enters only through the ionic strength $I {:}{=}\frac{1}{2} \sum _{i} \rho _i z_i^2$, where $\rho _i$ is the concentration and $z_i$ is the charge of ion-species *i*. For monovalent ions, as in the case of NaCl, the ionic strength reduces to the salt concentration, i.e., $I=\rho$. The salt concentration $\rho$ is expressed as molarity M, i.e., in units of mol/L, whereas the Debye screening length $\kappa ^{-1}$ and the Bjerrum length $\ell _B$ are conveniently expressed in Å. Note that the conversion factor for the length units *L*, in liters (0.001 m$^3$) versus Å, is absorbed into the numerical constant.

The temperature dependence of $\epsilon _r$ can be fitted from empirical data. In the current implementation we use the function proposed in [35]:

13 $$\begin{aligned} \epsilon _r(T) = 5321 T^{-1} + 233.76 - 0.9297T + 1.417\times 10^{-3}T^2 - 0.8292\times 10^{-6} T^3 \end{aligned}$$

with temperature in Kelvin. The temperature-dependence of $\epsilon _r$ ensures that the Bjerrum length $\ell _B$ is longer than the backbone bond length $l_{ss}$ in the entire temperature range, see Additional file 1: Fig. S3. The nonlinear electrostatic effects on unpaired bases thus become $\tau _{ss}=1/\ell _B$ for the monovalent cations considered here.

**Table 1:** RNA and DNA specific parameters

Parameter	Symbol [units]	RNA	DNA
Helical rise length	$l_{ds}$ [Å]	2.8	3.4
Backbone bond length	$l_{ss}$ [Å]	6	6.76
Slope of duplex init	*a* [kcal/mol]	$-0.45324$	$-0.58389$

The model of Einert et al. [20] is equally applicable to RNA and DNA. The only difference are the two geometric parameters and the empirically determined slope for the salt correction of the duplex initialization energy, which are summarized in Table 1. The values of two geometric parameters, helical rise per base and backbone bond length (phosphate-to-phosphate distance), are obtained from [17, 36–38]. Fitting data reported in [39] yields the slope for the salt correction for DNA duplex initialization, see Additional file 1: Fig. S4 for more detail. Our implementations allow for adapting the values of the two geometry parameters $l_{ss}$ and $l_{ds}$.

## Comparison with experimental data

### Available data sets

Even though the dependence of RNA structures on salt concentrations is of considerable practical interest, systematic data sets suitable for benchmarking salt correction models are by no means abundant. Most of the direct evidence for the salt dependence derives from studies of short duplexes and hairpins. Here, we analyzed datasets of melting experiments from four publications that reported melting temperature $T_m$ and/or free energy as well as corresponding enthalpy and entropy. The melting temperature is defined as the temperature at which half of the RNA molecules form duplexes or hairpins, respectively.

1. $18$ RNA self-complementary perfect duplexes of length $6$ or $8$ at the $5$ different sodium concentrations, 0.071, 0.121, 0.221, 0.621, and 1.021 M, with different species concentration $c$ were reported in [11, 40]. Free energies at $37 ^\circ C$ were obtained from $1/T_m$ vs. $\ln c$ plots. The data were also used by the same lab [40] to obtain optimised thermodynamic parameters, entropy and enthalpy, at a given salt concentration.
2. A different set of $8$ self-complementary duplexes, including two imperfect duplexes, of length $10$, $12$, and $14$ was reported in [41]. The data set covers two different species concentration $c$, $100\text { }\mu$M and $2\text { }\mu$M and two sodium concentrations, $1.0002$ M and $0.0102$ M.
3. Two hairpins at sodium concentrations of $0.021$, $0.036$, $0.061$, $0.111$, $0.211$, and $1.011$ M were reported in [42]. The hairpins consisted of a helix of length $5$ enclosing a hairpin loop of size $m=8$ or $10$.
4. $14$ hairpins at two salt concentrations, $1.02$ and $0.03$ M Na$^+$, measured in [43]. One of the hairpins consists of a helix of length $8$ and a hairpin loop of size $m=4$, while in the remaining 13 hairpins the helix is interrupted by a 1 nt bulge.

The values of melting temperatures, enthalpies, and entropies for datasets $2$, $3$, and $4$ were obtained by means of fitting melting curves in the respective publications.

### Comparison of duplex free energies

**Fig. 5:**
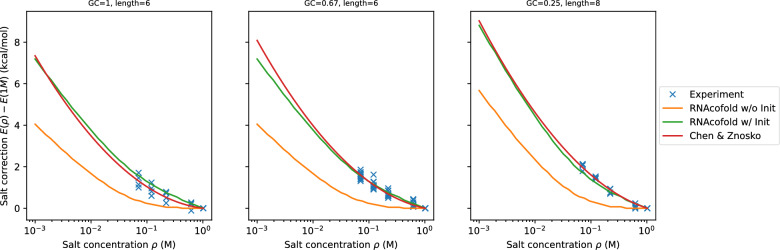
Duplex salt correction at different salt concentration from experiment (blue), Chen & Znosko (red), and ViennaRNA prediction with (green) and without (orange) salt correction for duplex initialization. The experimental free energy is derived from van ’t Hoff plots of $1/T_m$ versus $\ln c$. Most of the experimental values at 1 M are taken from [44]

Free energies were computed using RNAcofold [45, 46] with a self-complementary correction of $RT \ln 2$ added for all sequences that coincided with their reverse complement. Since the predicted free energy at the standard condition differs from the experimental values, we are interested in comparing the salt correction $g_w(\rho )=E_w(\rho ) - E_w(\rho _0)$ at concentration $\rho$ from the standard concentration $\rho _0=1.021$ M, where $E_w(\rho )$ is the free energy of duplex $w$ at concentration $\rho$. Let $l_w$ and $\text {gc} _w$ be the length and the fraction of GC of duplex $w$. Then the salt correction for RNAcofold is then given by

14 $$\begin{aligned} g_w^{\texttt {RNAcofold}}(\rho ) = (l_w-1)g_{\text {p}}(\rho ) + g_{\text {init}}(\rho ). \end{aligned}$$

The salt correction proposed by Chen & Znosko [11] is

15 $$\begin{aligned} g_w^{\text {C}} & {\text{Z}}(\rho ) = (0.324{\text{gc}}_w-0.486)\ln (\rho /\rho _0)+0.133(\ln (\rho /\rho _0))^2.\end{aligned}$$

The computational results are summarized in Fig. 5. Not surprisingly, the Chen & Znosko salt correction provides a slightly better fit to the data because the empirical formula for $g_w^{C \& Z}$ was obtained by fitting to the same data set. In contrast, only the duplex initialization $g_{\text {init}}(\rho )$ is fitted in our model. The largest deviations are observed for GC-only sequences.

### Comparison of duplex melting temperatures

Let *A* be a self-complementary RNA sequence and *AA* the corresponding dimer. The dimerization reaction is $2A \rightleftharpoons AA$. The corresponding concentrations are denoted by [*A*] and [*AA*], respectively. In equilibrium, we have

16 $$\begin{aligned} \dfrac{[AA]}{[A][A]} = \dfrac{Z_{AA}}{Z_AZ_A} = e^{(2G_A-G_{AA})/RT} \end{aligned}$$

where $Z_A$ and $Z_{AA}$ are the partition functions of the monomer and the dimer, respectively. Here $G_A=-RT\ln Z_A$ and $G_{AA}=-RT\ln Z_{AA}$ are the *ensemble* free energies of *A* and *AA*, respectively. Note that in a pure two-state system, we can replace $G_A$ and $G_{AA}$ by the corresponding minimum free energies $E_A$ and $E_{AA}$, respectively. We define the melting temperature $T_m$ as the temperature at which half of *A* forms the dimer *AA*, i.e., where $[AA]=c/4$ and $[A]=c/2$. Eq. (16) then yields

17 $$\begin{aligned} T_m = \dfrac{2G_A(T_m)-G_{AA}(T_m)}{-R\ln c}, \end{aligned}$$

where we write $G_A(T_m)$ and $G_{AA}(T_m)$ to emphasize the temperature dependence of the ensemble free energies. The ensemble free energy $G_{AA}$ for the pure dimer state is accessible directly via the function vrna_pf_dimer() within the ViennaRNA library. The correction for self-complementary is already taken into account during the computation of the partition function [46]. Since the ensemble free energy is also a function of temperature, we use a binary search to find the melting temperature $T_m$.

**Fig. 6:**
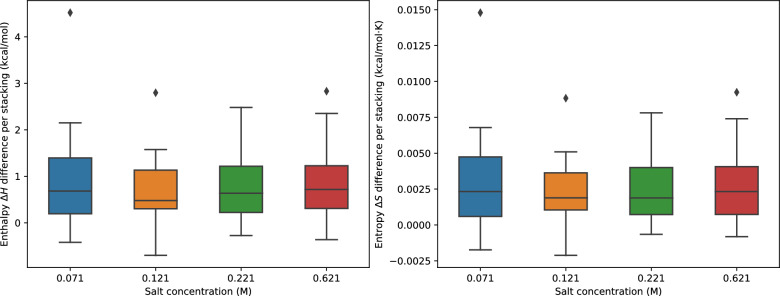
Boxplot of differences between experimental and predicted salt effects for 18 short duplexes measured in [11]. Enthalpies and entropies were estimated from linear regression according to van ’t Hoff’s Eq. (18) from the data plotted in Additional file 1: Fig. S5

For the data set comprising 18 short duplexes, experimental melting temperatures are available for several distinct species concentrations $c$. Van ’t Hoff’s equation

18 $$\begin{aligned} \dfrac{1}{T_m} = \dfrac{R}{\Delta H}\ln c + \dfrac{\Delta S}{\Delta H} \end{aligned}$$

implies a linear relationship between changes in $1/T_m$ and changes in RNA concentration. We compare the predicted and experimental “van ’t Hoff” plots, i.e., diagrams of $1/T_m$ *versus* $\ln c$, see Additional file 1: Fig. S5. Overall, we observe an excellent agreement on the slope between RNAcofold prediction and experiment. Predicted and experimental intercepts are slightly more different for a few of the duplexes. To further investigate, we performed linear regression on each van ’t Hoff plot and obtained the entropy and the enthalpy of each duplex at different salt concentration using Eq. 18. Figure 6 shows the difference of these thermodynamic values per base pair stack computed from the predictions and from the experiments. For both enthalpy and entropy, the predicted values are in general larger than the experimental one, but are in the same order of magnitude. The differences in enthalpy and entropy largely compensate in the free energy $\Delta G=\Delta H-T\Delta S$.

**Fig. 7:**
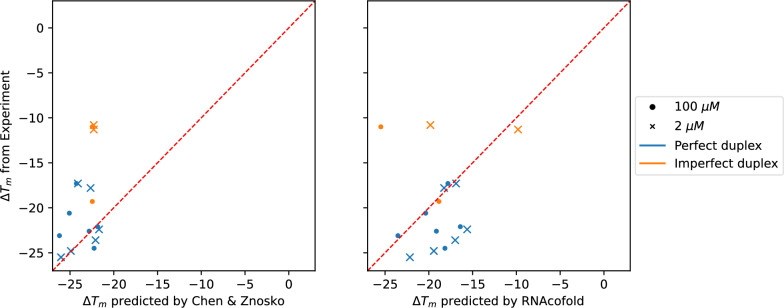
Comparison of experimental and predicted melting temperature corrections $\Delta T_m$ using the empirical fit by Chen & Znosko [11] (left) and RNAcofold with the salt corrections terms described in the present contribution (right). The data of perfect duplex is plotted in blue while the one of imperfect duplex is in orange. The red dashed line draws $x=y$ meaning the predicted value matches the experimental one

For the second dataset consisting of longer duplexes, we are interested in melting temperature correction $\Delta T_m (0.01\text {M})$ from the standard condition $1$ M since the data is only available for two species concentrations. Chen & Znosko [11] proposed the following fit

19 $$\begin{aligned} \Delta T_m ^{C \& Z}(\rho ) = (-1.842\text {gc} _w+2.675)\ln \rho -0.7348(\ln \rho )^2 \end{aligned}$$

where $\text {gc} _w$ is the GC fraction of duplex $w$. Figure 7 shows the experimental melting temperature correction compared with $\Delta T_m ^{C \& Z}$ and the one computed by RNAcofold. Overall, the salt corrections described above show similar agreement (Root mean squared deviation $r=5.638$) with the experiment as the empirical fit ($r=5.862$) from [11].

### Comparison of hairpin free energies

**Fig. 8:**
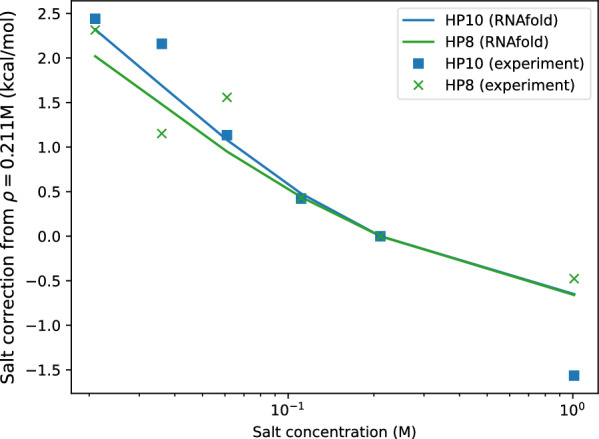
Comparison of free energy correction from experiment for hairpin HP8 (green) and HP10 (blue) with RNAfold prediction

For the hairpins HP8 and HP10 described in [42], entropy, enthalpy, and free energy were derived from melting experiments. For comparison, we computed the free energy using RNAfold at $37^\circ C$ for different salt concentrations $\rho$. Figure 8 summarizes the experimental and predicted free energy corrections from 0.211 M. They are in very good agreement in particular for lower salt concentrations.

For the 14 hairpins measured in [43] we used RNAfold to compute the free energy difference between salt concentration 0.03 M and 1.02 M at $37^\circ C$. For the hairpin with helix length 8, the free energy difference is 2.9 kcal/mol from experiment and 1.94 kcal/mol from computation. For the remaining 13, structurally identical, hairpins, the average experimental free energy difference is $3.68\pm 0.30$ kcal/mol compared to a predicted value at 2.15 kcal/mol. In general, the model under-estimates the salt correction by 1.5 kcal/mol.

## Discussion

Salt concentrations significantly influence folding and thermodynamics of nucleic acids. In this contribution we report on the implementation of an approximate physical model proposed by Einert and Netz [20] that represent the RNA backbone as a charged polymer interacting by means of a Debye-Hückel potential. It is worth noting that model has no parameter that captures individual properties of the monovalent cation. It therefore applies equally to $\hbox {Na}^{+}$, for which experimental data were available for comparison, and other monovalent cations. The model was adapted here to preserve the linear multi-loop model required for computational efficiency and extended by a empirical initiation term for duplex formation. While not perfect, the model reproduces experimental thermodynamic data on the NaCl dependence of folding energies and melting temperatures with reasonable accuracy. We note that the salt-dependent energies for stacks diverge logarithmically for vanishing salt concentrations $\rho$. The model thus cannot be used if cations are virtually absent.

The approach taken here has the practical advantage that it does not require any changes in the folding algorithms. The modification of the energy parameters is sufficient. The ViennaRNA packages handles this step during preprocessing. As a consequence, the computational performance of the folding routines remain unchanged. Moreover, the salt corrections are consistently applied to all variants of the folding algorithms, e.g. minimum energy and partition function computations, consensus computations from alignments, and co-folding of two or more components.

**Fig. 9:**
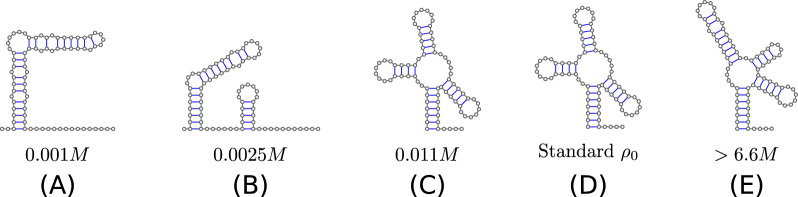
Examples of structural transitions. MFE structures of a tRNA sequence at different salt concentrations are predicted with RNAfold. Within the concentration range from 0.011 to 6.6 M, the MFE structure is same as the one at the standard condition (**C**, **D**). The denaturation is observed at low concentration (**A**, **B**), while at high concentration ($>6.6$ M, corresponding to a saturated saline solution), **E** become the MFE structure. The tRNA sequence used is GCGGAUUUAGCUCAGUUGGGAGAGCGCCAGACUGAAGAUCUGGAGGUCCUGUGUUCGAUCCACAGAAUUCGCACCA

**Fig. 10:**
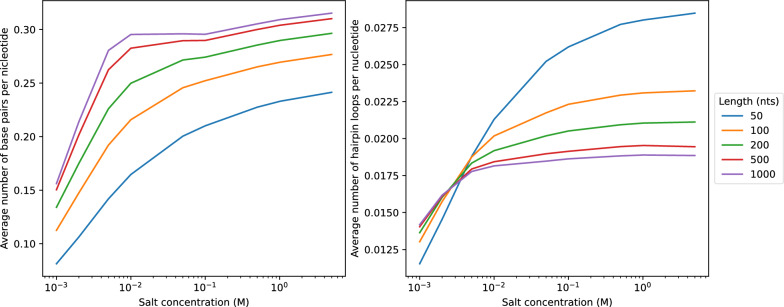
Average number of base pairs (left) and hairpin loops (right) per nucleotides in MFE structures at different salt concentrations. For each length $n$, $5~000$ RNA sequences are uniformly and randomly selected from $\{A,C,G,U\}^n$. Each sequence is then folded using RNAfold at different salt concentrations

Changes in salt concentration also affect the predicted secondary structures, see Fig. 9 for an example. A quantitative analysis, Fig. 10, confirms that the number of base pairs increases with salt concentration. Interestingly, the number of hairpin loops as measure for the overall “branched-ness” of the secondary structure, also increases. To our knowledge, there are no detailed experimental data that document structural changes as a function of NaCl concentration so that a directed validation of structures predicted for low salt concentrations remains a topic for future research.

**Fig. 11:**
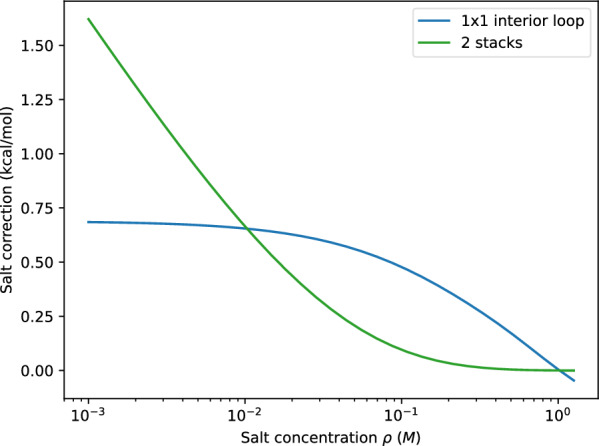
Free energy correction of an 1$\times$1 interior loop (blue) and a helix of two stackings (green) at different salt concentration

Additional file 1: Fig. S6 shows the salt correction comparison with the coarse grained model of Tan & Chen for duplex [16] and hairpin loops [17]. Both models give similar predictions for duplexes of length 6 to 10. However, the Tan & Chen correction is not linear in length making it hard to be integrated into the current folding grammar. On the other hand, salt corrections for hairpin loops computed using two models have different behaviors, where the Tan & Chen correction diverges with increasing loop size.

In the current model, a basepair mismatch in a helix is treated as an 1$\times$1 interior loop and thus is associated with the salt correction for loops at non-standard salt concentrations. However, such a mismatch is likely to result in a slightly distorted helix that could still be seen as two parallel charged rods. One could therefore argue, that the salt correction for 2 stacked pairs rather than for a loop should be applied. Figure 11 shows the difference of these two cases as a function of salt concentration. To our knowledge, there are no experimental data that could be used to decide which approach is more appropriate.

The approach taken here does not account for all effects of ions on RNA folding. Most importantly, it covers only unspecific interactions and thus does not describe specific interactions e.g. of $\hbox {Mg}^{+}$ with specific binding sites. Even for unspecific interactions, the validity of the model can be argued stringently only for mono-valent ions [20].

We note that the empirical salt-correction formula for stacking energies proposed in [7] incorporates the combined salt concentrations as a linear combination $[{\text {Na}}^{+}]+3.3[{\text {Mg}}^{2+}]$. In essence this term expresses the cationic contribution to the ionic strength (up to the empirically determined coefficient 3.3 instead of the theoretical value 4). This may be taken as a hint that the model implemented here may also serve as reasonable approximation for more mixtures ions. At present, available data, e.g. [47], are not sufficient to test whether replacing $\rho$ by the ionic strength is sufficient to reasonably account for mixtures of mono-valent and di-valent cations.

The model described here does not account for salt effects of RNA structures that are neither loops nor stacked base pairs. In particular it does not apply to G quadruplexes [48], which optionally can be included in secondary structure predictions [49]. Separate models for the ion dependencies of such features will need to be derived that account e.g. for the $\hbox {K}^{+}$-dependent stabilization of RNA quadruplexes.

## Supplementary Information

**Additional file 1: Figure S1.** Length distribution of multi-loops. Distribution of multi-loop size *L*, number of backbone bonds, among MFE structures of 5000 uniformly selectedsequences at varied length. **Figure S2.** Approximation Error for *K*_0_. In [20] an approximation for the difference of *K*_0_ at a given concentration and 1 M was proposed. However, wenoticed that this approximation yields a non-vanishing salt correction at 1 M. We therefore used the Cephes libraryto compute *K*_0_ directly. The panel shows the salt correction of base pair stack at 37 °C in the function of saltconcentration using the approximation (blue) and the precise computation implemented in ViennaRNA (orange). **Figure S3**. Nonlinear electrostatic effects τss. In [20], the permittivity (relative dielectric constant) *ε*_r_ of water *ε*_r_ ≈ 80 is assumed to be temperatureindependent. This assumption results in a discontinuity of τss at around 53.3 °C. Incorporating the empiricaltemperature dependence of *ε*_r_ in eq. (13) [35] results in 1/ℓB < 1/lss. **Figure S4.** Comparison of experimental and predicted melting temperature corrections Δ*T*_m_ of DNA duplexes. In [39], the authors performed melting experiment of DNA duplexes at different salt concentrations (Table 2) andcollected an independent set of DNA melting temperatures (Table 5). The former one is used to fit the saltcorrection for DNA duplex initialization and the later one is used for validation. Only the duplexes with lengthsmaller than 11 are used in both datasets. **Figure S5**. Van t’Hoff plots for 18 duplexes. Plotting 1/Tm versus ln c shows a generally good agreement of between predictions and the experimental datafrom from [11]. **Figure S6.** Salt correction comparison with Tan & Chen model.Comparison of salt correction predicted with ViennaRNA for hairpin loop and duplex of different size with the onespredicted with Tan & Chen model [16, 17]. **Table S1.** Numeric values of physical constants.

## Data Availability

The implementation is available in the ViennaRNA Package starting with version 2.6.0 available at https://www.tbi.univie.ac.at/RNA. The tutorial-like notebook and the data to produce all figures are available at https://github.com/ViennaRNA/salty.
