# Supplementary material for: Mono-valent salt corrections for RNA secondary structures in the ViennaRNA package
Source: Algorithms Mol Biol. 2023 Jul 29;18:8. doi: 10.1186/s13015-023-00236-0 (PMC10386259; doi:10.1186/s13015-023-00236-0)
Supplement: Supplementary file 1 — Additional file 1: Figure S1. Length distribution of multi-loops. Distribution of multi-loop size L, number of backbone bonds, among MFE structures of 5000 uniformly selectedsequences at varied length. Figure S2. Approximation Error for K0. In [20] an approximation for the difference of K0 at a given concentration and 1 M was proposed. However, wenoticed that this approximation yields a non-vanishing salt correction at 1 M. We therefore used the Cephes libraryto compute K0 directly. The panel shows the salt correction of base pair stack at 37 °C in the function of saltconcentration using the approximation (blue) and the precise computation implemented in ViennaRNA (orange). Figure S3. Nonlinear electrostatic effects τss. In [20], the permittivity (relative dielectric constant) εr of water εr ≈ 80 is assumed to be temperatureindependent. This assumption results in a discontinuity of τss at around 53.3 °C. Incorporating the empiricaltemperature dependence of εr in eq. (13) [35] results in 1/ℓB < 1/lss. Figure S4. Comparison of experimental and predicted melting temperature corrections ΔTm of DNA duplexes. In [39], the authors performed melting experiment of DNA duplexes at different salt concentrations (Table 2) andcollected an independent set of DNA melting temperatures (Table 5). The former one is used to fit the saltcorrection for DNA duplex initialization and the later one is used for validation. Only the duplexes with lengthsmaller than 11 are used in both datasets. Figure S5. Van t’Hoff plots for 18 duplexes. Plotting 1/Tm versus ln c shows a generally good agreement of between predictions and the experimental datafrom from [11]. Figure S6. Salt correction comparison with Tan & Chen model.Comparison of salt correction predicted with ViennaRNA for hairpin loop and duplex of different size with the onespredicted with Tan & Chen model [16, 17]. Table S1. Numeric values of physical constants. [file 13015_2023_236_MOESM1_ESM.pdf]

Mono-valent salt corrections for RNA secondary structures in the ViennaRNA package

Supplementary Data

Hua-Ting Yao, Ronny Lorenz, Ivo L. Hofacker, Peter F. Stadler

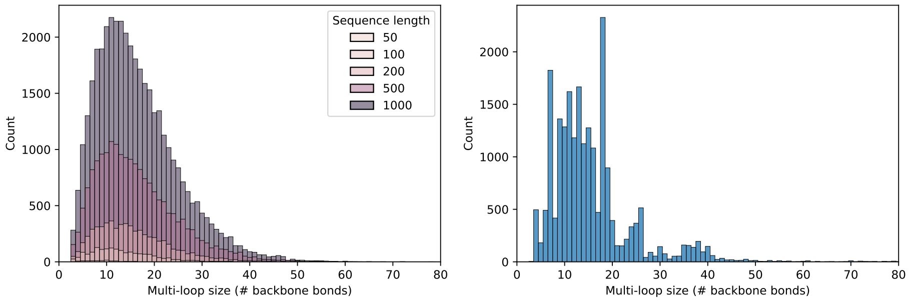

Figure S1: Size distribution of multi-loops. Distribution of multi-loop size L, number of backbone bonds, among predicted MFE structures (left) and natural RNA structures (right). The MFE structure is computed with `RNAfold` at standard condition on 5 000 uniformly and randomly selected sequences from $\{A, C, G, U\}^n$ for each length $n \in \{50, 100, 200, 500, 1\ 000\}$. In total, 3 582 natural RNA secondary structures featuring multi-loops is downloaded from RNA STRAND database [1]. The distribution is drawn with outlier removal, multi-loop size larger than 80.

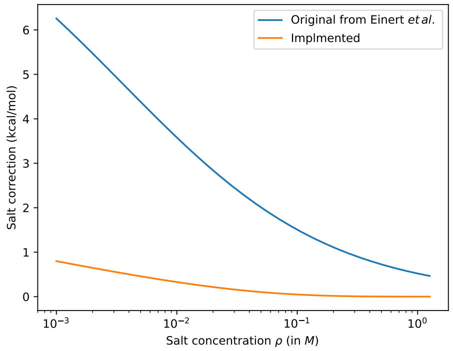

Figure S2: Approximation Error for K_0. In [2] an approximation for the difference of K_0 at a given concentration and $1M$ was proposed. However, we noticed that this approximation yields a non-vanishing salt correction at $1M$. We therefore used the Cephes library to compute K_0 directly. The panel shows the salt correction of base pair stack at $37°C$ in the function of salt concentration using the approximation (blue) and the precise computation implemented in `ViennaRNA` (orange).

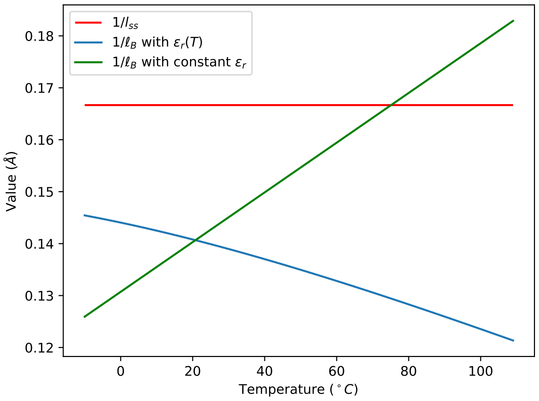

Figure S3: Nonlinear electrostatic effects τ_{ss}. In [2], the permittivity (relative dielectric constant) ϵ_r of water $\epsilon_r \approx 80$ is assumed to be temperature independent. This assumption results in a discontinuity of τ_{ss} at around $75\,^\circ C$. Incorporating the empirical temperature dependence of ϵ_r results in $1/\ell_B < 1/l_{ss}$.

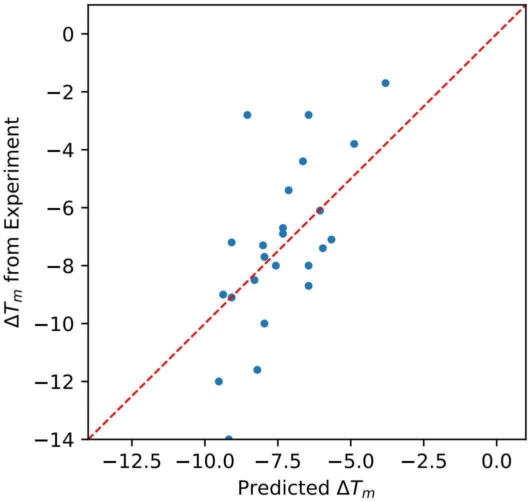

Figure S4: Comparison of experimental and predicted melting temperature corrections ΔT_m of DNA duplexes. Experimental DNA duplex melting temperature data is from Table 5 in [3]. Note that only the duplexes with length smaller or equal to 11 are considered.

Due to the lack of DNA energy data at different salt concentrations, the salt correction for DNA duplex initialization is fitted to melting temperature data. The melting temperature corrections from $1.02M$ for DNA duplexes of length 10 and 11 in Table 2 in [3] are used for fitting. Note that the DNA sequence used is not self-complementary. Let A and B be DNA sequences and AB the corresponding dimer. The dimerization reaction is $A + B \rightleftharpoons AB$. The corresponding concentrations are denoted by $[A]$, $[B]$ and $[AA]$, respectively. In equilibrium, we have

$$\frac{[AB]}{[A][B]} = e^{(G_A+G_B-G_{AB})/RT} \tag{1}$$

where G_A, G_B, and G_{AB} are the *ensemble* free energies of A, B, and AA, respectively. The melting temperature T_m is the temperature at which half of A and B form the dimer AB, i.e., where $[A] = [B] = [AA] = c/4$ with c is the initial concentration of the single strand. Equ.(1) then yields

$$T_m = \frac{G_A(T_m) + G_B(T_m) - G_{AA}(T_m)}{R\ln 4 - R\ln c}, \tag{2}$$

The fitting yields the salt correction for DNA duplex initialization

$$g_{\text{init}}(\rho) = a\ln(\frac{\rho}{\rho_0}) \qquad \text{with} \quad a = -0.58389\text{kcal/mol}. \tag{3}$$

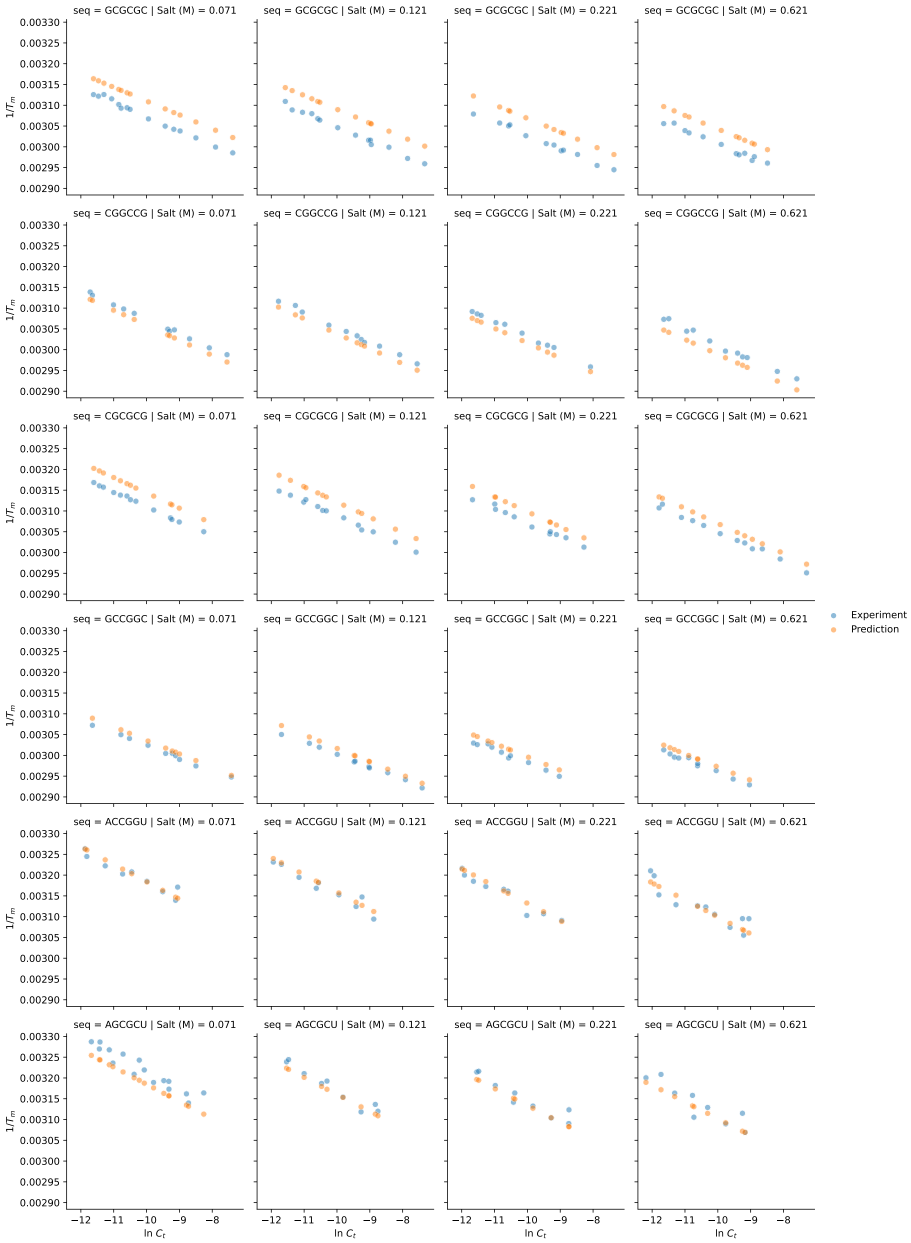
seq = GCGCGC | Salt (M) = 0.071
seq = GCGCGC | Salt (M) = 0.121
seq = GCGCGC | Salt (M) = 0.221
seq = GCGCGC | Salt (M) = 0.621
seq = CGGCCG | Salt (M) = 0.071
seq = CGGCCG | Salt (M) = 0.121
seq = CGGCCG | Salt (M) = 0.221
seq = CGGCCG | Salt (M) = 0.621
seq = CGCGCG | Salt (M) = 0.071
seq = CGCGCG | Salt (M) = 0.121
seq = CGCGCG | Salt (M) = 0.221
seq = CGCGCG | Salt (M) = 0.621
seq = GCCGGC | Salt (M) = 0.071
seq = GCCGGC | Salt (M) = 0.121
seq = GCCGGC | Salt (M) = 0.221
seq = GCCGGC | Salt (M) = 0.621
seq = ACCGGU | Salt (M) = 0.071
seq = ACCGGU | Salt (M) = 0.121
seq = ACCGGU | Salt (M) = 0.221
seq = ACCGGU | Salt (M) = 0.621
seq = AGCGCU | Salt (M) = 0.071
seq = AGCGCU | Salt (M) = 0.121
seq = AGCGCU | Salt (M) = 0.221
seq = AGCGCU | Salt (M) = 0.621
$1/T_m$
ln C_t
Experiment
Prediction

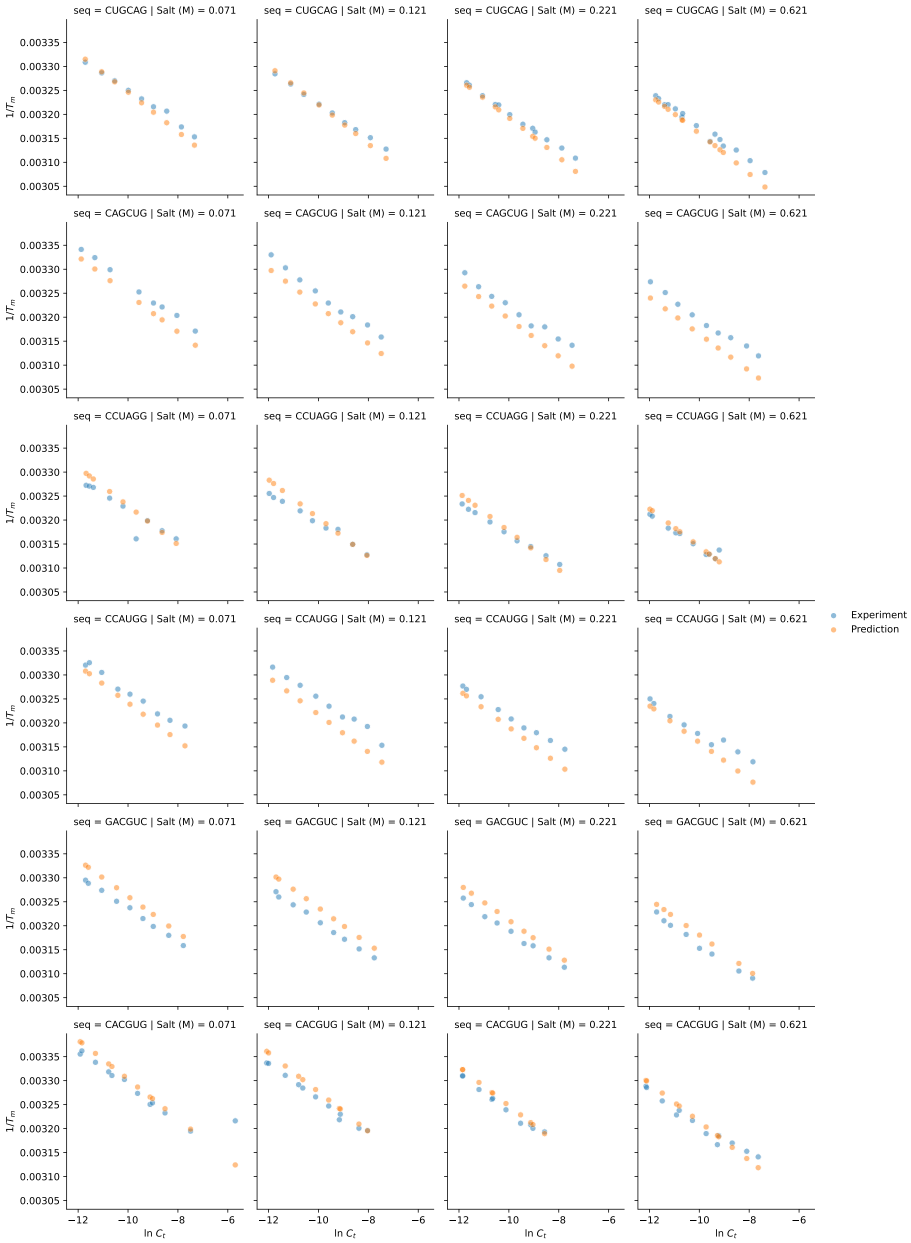
seq = CUGCAG | Salt (M) = 0.071
seq = CUGCAG | Salt (M) = 0.121
seq = CUGCAG | Salt (M) = 0.221
seq = CUGCAG | Salt (M) = 0.621
seq = CAGCUG | Salt (M) = 0.071
seq = CAGCUG | Salt (M) = 0.121
seq = CAGCUG | Salt (M) = 0.221
seq = CAGCUG | Salt (M) = 0.621
seq = CCUAGG | Salt (M) = 0.071
seq = CCUAGG | Salt (M) = 0.121
seq = CCUAGG | Salt (M) = 0.221
seq = CCUAGG | Salt (M) = 0.621
seq = CCAUGG | Salt (M) = 0.071
seq = CCAUGG | Salt (M) = 0.121
seq = CCAUGG | Salt (M) = 0.221
seq = CCAUGG | Salt (M) = 0.621
seq = GACGUC | Salt (M) = 0.071
seq = GACGUC | Salt (M) = 0.121
seq = GACGUC | Salt (M) = 0.221
seq = GACGUC | Salt (M) = 0.621
seq = CACGUG | Salt (M) = 0.071
seq = CACGUG | Salt (M) = 0.121
seq = CACGUG | Salt (M) = 0.221
seq = CACGUG | Salt (M) = 0.621
$1/T_m$
0.00335
0.00330
0.00325
0.00320
0.00315
0.00310
0.00305
−12
−10
−8
−6
ln C_t
Experiment
Prediction

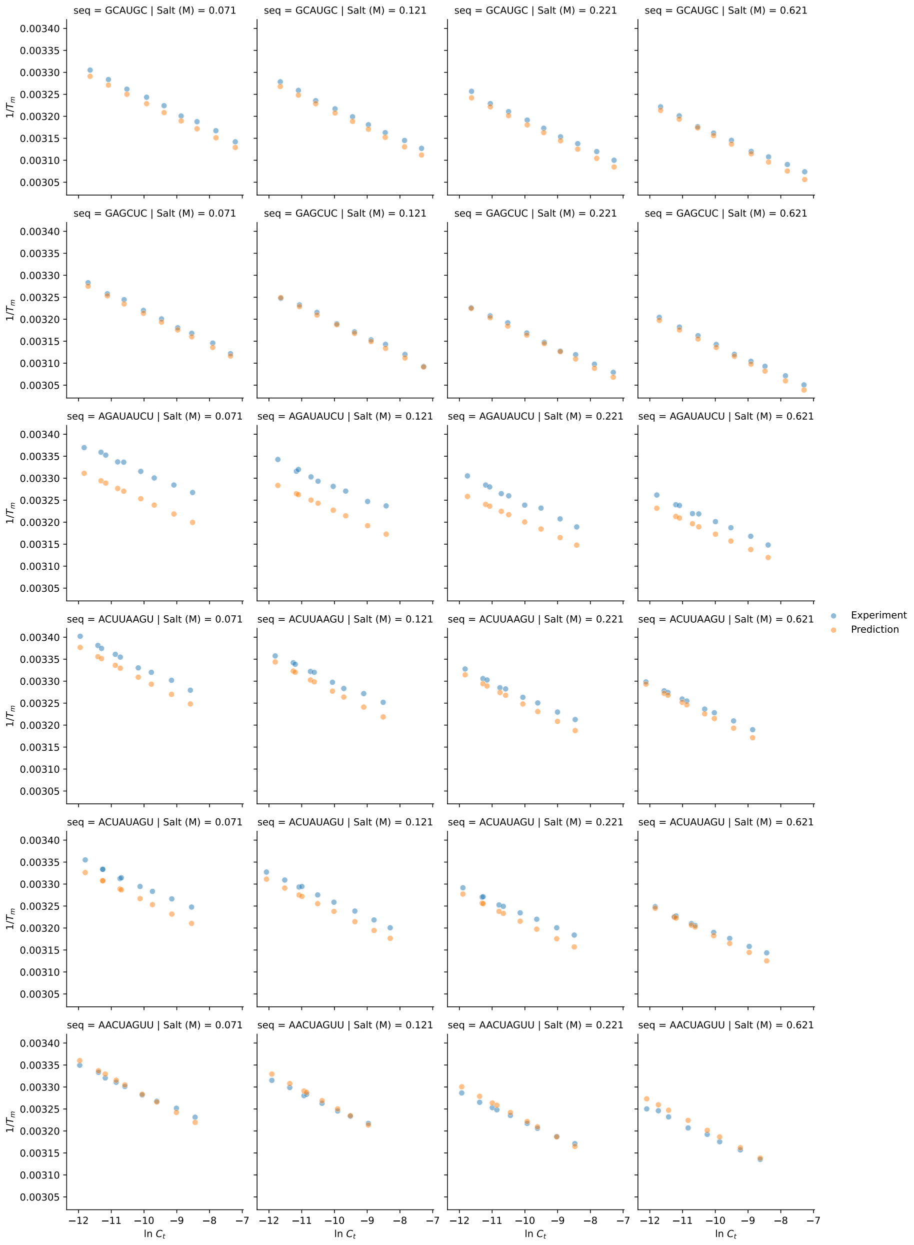

Figure S5: Van t' Hoff plots for 18 duplexes. Plotting $1/T_m$ versus $\ln c$ shows a generally good agreement of between predictions and the experimental data from [4, 5].

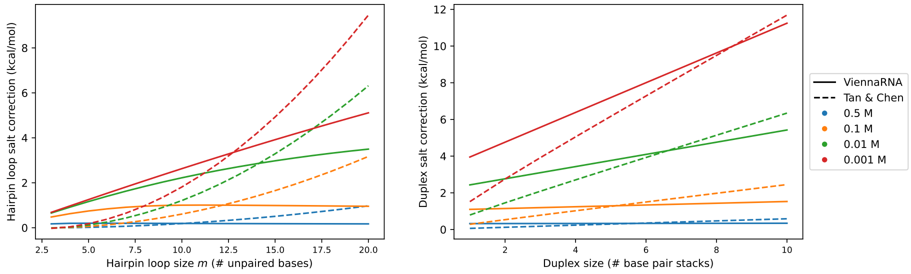

Figure S6: Comparison of salt correction for hairpin loop (left) and duplex (right) with Tan & Chen model (dashed). The salt correction for duplex initialization is included in the one for duplex computed with ViennaRNA. For Tan & Chen model, the correction for hairpin loop is computed using Equ.(9) of [6] with the end-to-end distance $x = 17\,\mathring{A}$, while the one for duplex is from Equ.(13) in [7].

Table S1: Numeric values of physical constants.

Constant	Symbol [units]	Value
Unit charge	e [C]	$1.602 \cdot 10^{-19}$
Boltzmann constant	k_B [eV/K]	$8.617 \cdot 10^{-5}$
Vacuum permittivity	ϵ_0 [F/Å]	$8.854 \cdot 10^{-22}$
Avogadro constant	N_A [mol^{-1}]	$6.022 \cdot 10^{23}$

References

[1] Andronescu, M., Bereg, V., Hoos, H.H., Condon, A.: RNA STRAND: the RNA secondary structure and statistical analysis database. BMC Bioinformatics **9**, 340 (2008). doi:10.1186/1471-2105-9-340

[2] Einert, T.R., Netz, R.R.: Theory for RNA folding, stretching, and melting including loops and salt. Biophys. J. **100**, 2745–2753 (2011). doi:10.1016/j.bpj.2011.04.038

[3] Owczarzy, R., You, Y., Moreira, B.G., Manthey, J.A., Huang, L., Behlke, M.A., Walder, J.A.: Effects of sodium ions on DNA duplex oligomers: improved predictions of melting temperatures. Biochemistry **43**(12), 3537–3554 (2004). doi:10.1021/bi034621r

[4] Chen, Z., Znosko, B.M.: Effect of sodium ions on RNA duplex stability. Biochemistry **52**(42), 7477–7485 (2013). doi:10.1021/bi4008275

[5] Ferreira, I., Jolley, E.A., Znosko, B.M., Weber, G.: Replacing salt correction factors with optimized RNA nearest-neighbour enthalpy and entropy parameters. Chem. Phys. **521**, 69–76 (2019). doi:10.1016/j.chemphys.2019.01.016

[6] Tan, Z.-J., Chen, S.-J.: Salt dependence of nucleic acid hairpin stability. Biophys. J. **95**(2), 738–752 (2008). doi:10.1529/biophysj.108.131524

[7] Tan, Z.-J., Chen, S.-J.: RNA helix stability in mixed Na^+/Mg^{2+} solution. Biophys J **92**(10), 3615–3632 (2007). doi:10.1529/biophysj.106.100388
